# The Impact of Glycoengineering on the Endoplasmic Reticulum Quality Control System in Yeasts

**DOI:** 10.3389/fmolb.2022.910709

**Published:** 2022-06-02

**Authors:** Mari A. Piirainen, Alexander D. Frey

**Affiliations:** ^1^ Department of Bioproducts and Biosystems, Aalto University, Espoo, Finland; ^2^ Kemistintie 1, Aalto University, Otakaari, Finland

**Keywords:** protein N-glycosylation, endoplasmic reticulum associated protein degradation (ERAD), endoplasmic reticulum quality control (ERQC), yeast, glycoengineering

## Abstract

Yeasts are widely used and established production hosts for biopharmaceuticals. Despite of tremendous advances on creating human-type N-glycosylation, N-glycosylated biopharmaceuticals manufactured with yeasts are missing on the market. The N-linked glycans fulfill several purposes. They are essential for the properties of the final protein product for example modulating half-lives or interactions with cellular components. Still, while the protein is being formed in the endoplasmic reticulum, specific glycan intermediates play crucial roles in the folding of or disposal of proteins which failed to fold. Despite of this intricate interplay between glycan intermediates and the cellular machinery, many of the glycoengineering approaches are based on modifications of the N-glycan processing steps in the endoplasmic reticulum (ER). These N-glycans deviate from the canonical structures required for interactions with the lectins of the ER quality control system. In this review we provide a concise overview on the N-glycan biosynthesis, glycan-dependent protein folding and quality control systems and the wide array glycoengineering approaches. Furthermore, we discuss how the current glycoengineering approaches partially or fully by-pass glycan-dependent protein folding mechanisms or create structures that mimic the glycan epitope required for ER associated protein degradation.

## Introduction

Yeasts are widely used and established production hosts for biopharmaceuticals. *Saccharomyces cerevisiae* is by far the most utilized yeast species for therapeutic proteins and has been approved as a production host for 35 different products. Following by a large margin, *Pichia pastoris* is used for the production of three, while *Hansenula polymorpha* is utilized for producing two therapeutic proteins. The yeast-based biopharmaceuticals currently in market include various insulin products and vaccines, but also other peptide hormones, a blood clotting factor subunit and a recombinant urate oxidase (Walsh, 2018). So far, N-glycosylated biopharmaceuticals manufactured by yeasts are currently missing on the market.

The intricacy of the N-glycan biosynthesis pathways and the wide range of end products of this modification renders the engineering of glycosylation process very demanding. Despite the heterogeneity of N-glycan structures, the formation of a lipid-linked oligosaccharide (LLO), the transfer of the glycan to a protein, and the trimming of the N-glycan during protein folding are highly conserved processes and thus N-glycan maturation in the Golgi apparatus starts from the same structure.

The N-glycans fulfill several purposes, they modulate the properties of the protein product, support protein folding through binding to calnexin and export of folded glycoproteins and provide the necessary cues to the endoplasmic reticulum associated protein degradation (ERAD) machinery to initiate protein turnover. The currently applied glycoengineering approaches affect the N-glycan processing in the endoplasmic reticulum (ER) in various ways and the different engineering approaches have the potential to partially or fully sidestep glycan-dependent protein folding and disposal machineries. In this review, we summarize the current knowledge on N-glycan biosynthesis and glycan-dependent quality control systems in the context of glycoengineering in yeasts.

### The Endoplasmic Reticulum Protein N-Glycosylation System

**FIGURE 1:**
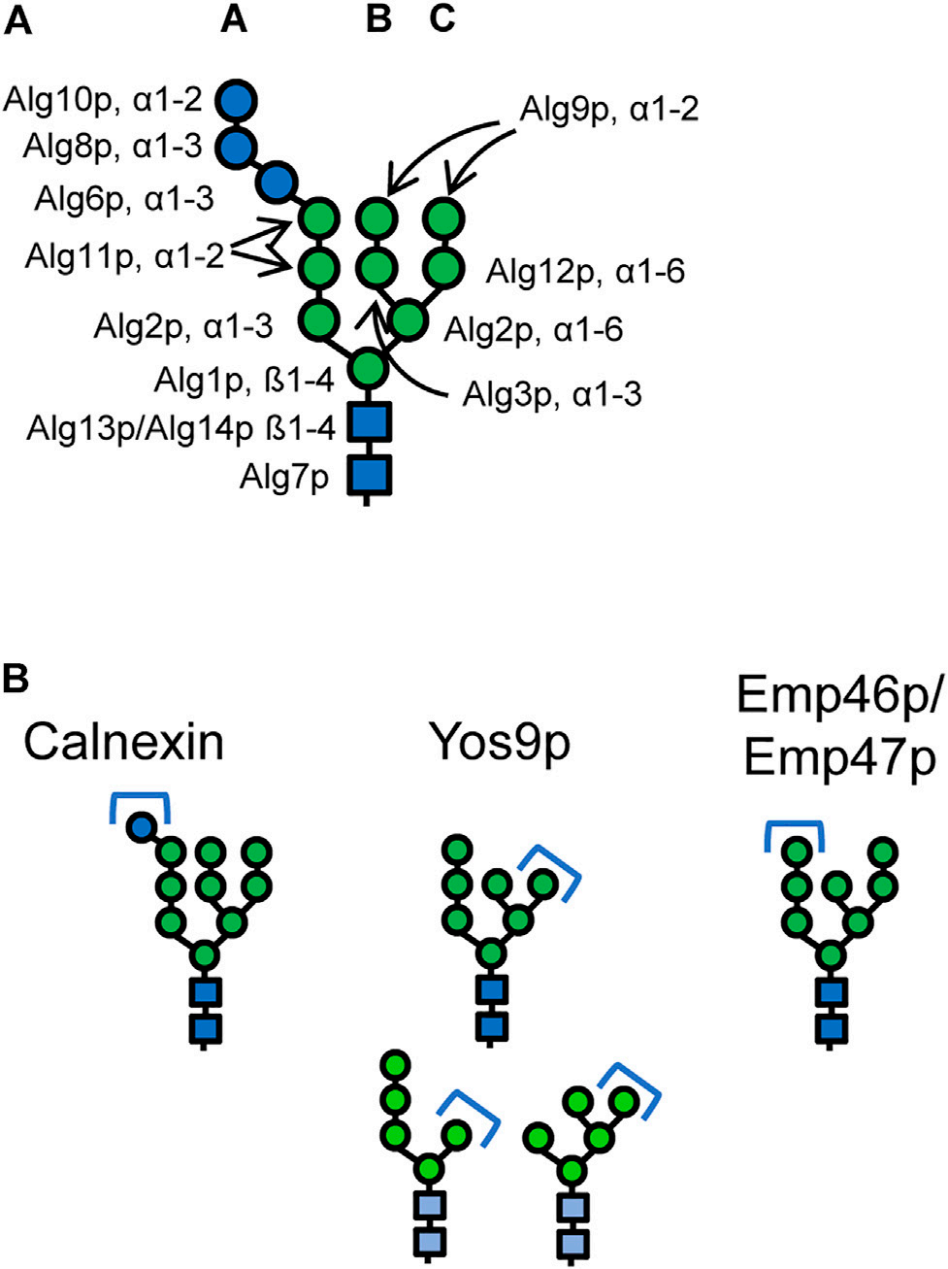
**(A)** Structure of the eukaryotic dolichol-linked oligosaccharide. Linkages and the names of the enzymes catalysing the respective steps are given. Deletion of *ALG11* gene prevents formation of the A-branch. Deletion of *ALG3* gene prevents formation the B- and C-branch mainly generating a Man_5_GlcNAc_2_ structure. Glucosylation of the A-branch is impaired in Δ*alg3* mutants. **(B)** Canonical N-glycan structures that mediate binding to the lectins calnexin/calreticulin, the ERAD lectin Yos9p, and the ER export receptors Emp46p/Emp47p, respectively. Alternative Yos9p substrates are marked in lighter hues. Blue circles: glucose, green circles: mannose, blue squares: N-acetylglucosamine.

The LLO assembly that is initiated on the cytoplasmic side of the ER leads in a stepwise process to the formation of the Glc_3_Man_9_GlcNAc_2_ oligosaccharide (Figure 1A). First, a phosphorylated N-acetylglucosamine is transferred to a dolichol phosphate by Alg7p (Barnes et al., 1984; Kukuruzinska and Robbins, 1987). A second N-acetylglucosamine is then added via a β1-4 bond by an UDP-GlcNAc transferase complex consisting of Alg13p and Alg14p (Bickel et al., 2005; Chantret et al., 2005). The formed Dol-P-P-GlcNAc_2_ structure receives three mannose residues by Alg1p and Alg2p leading to the formation of the branched core glycan structure comprising terminal α1-3 linked and α1-6 linked mannoses (Huffaker and Robbins, 1982; Couto et al., 1984; O’Reilly et al., 2006). Then, Alg11p adds two α1-2 linked mannose residues to the α1-3 arm of the LLO initiating the A branch (Cipollo et al., 2001; O’Reilly et al., 2006). The resulting Man_5_GlcNAc_2_-P-P-Dol is translocated to the luminal side of the ER. Efforts to identify the flippase and elucidate the mechanism of the translocation reaction through *in vivo* and *in vitro* experiments produced conflicting results and the identity of the flippase and the specific role of Rft1p remains unresolved (Helenius et al., 2002; Frank et al., 2008; Sanyal et al., 2008; Rush et al., 2009; Verchère et al., 2021).

On the luminal side of the ER, the LLO precursor is further branched and elongated. The α1-6 linked arm of the LLO receives an α1-3 linked mannose by Alg3p (Huffaker and Robbins, 1983; Aebi et al., 1996b; Sharma et al., 2001) forming the B branch which is capped with an α1-2 linked mannose by Alg9p (Burda et al., 1996; Frank and Aebi, 2005). A third branch, C branch, is formed as Alg12p transfers an α1-6 linked mannose to the α1-6 arm of the core glycan (Burda et al., 1999). Again Alg9p catalyzes the addition of a terminal mannose (Frank and Aebi, 2005). The final steps of the LLO biosynthesis include the addition of three glucose residues to the A branch. The first two glucoses are added via α1-3 linkages by Alg6p and Alg8p and the third via an α1-2 linkage by Alg10p, respectively (Runge and Robbins, 1986; Stagljar et al., 1994; Reiss et al., 1996; Burda and Aebi, 1998).

The complete Glc_3_Man_9_GlcNAc_2_ oligosaccharide is transferred *en bloc* via an N-glycosidic bond to an asparagine residue in the acceptor sequon Asn-X-Ser/Thr by the oligosaccharyltransferase (OST) complex. To ensure that only completely assembled LLOs are transferred, the OST in most eukaryotes has a strong preference for the Glc_3_Man_9_GlcNAc_2_ LLO, and the outermost glucose together with the chitobiose stem (GlcNAc_2_) of the LLO are important determinants for the substrate recognition (Burda et al., 1999).

### N-Glycan Trimming Reactions Involved in Protein Folding and Degradation

The following glycan trimming reactions play important roles in protein folding and marking of misfolded proteins for ERAD. First, two glucose residues are very rapidly removed from the Glc_3_Man_9_GlcNAc_2_ glycan (Jakob et al., 1998). The resulting Glc_1_Man_9_GlcNAc_2_ glycan is specifically recognized by the lectin calnexin (Cne1p) (Parlati et al., 1995; Xu et al., 2004b) (Figure 1B). Removal of the last glucose residue of the N-glycan by the glucosidase heterodimer Gls2p/Gtb1p releases the protein from calnexin (Trombetta et al., 1996; Wilkinson et al., 2006). In some yeasts, an α1-3 linked glucose residue can be re-transferred to the Man_9_GlcNAc_2_ glycans by a UDP-glucose:glycoprotein glucosyltransferase (UGGT) enabling rebinding to calnexin and extending the time to correctly fold. However for example, *S. cerevisiae* lacks UGGT (Fernández et al., 1994), making the removal of the last glucose residue an irreversible process. After the removal of the glucose residues, one mannose residue is removed from the B branch of the N-glycan by the α1-2 mannosidase Mns1p (Camirand et al., 1991). Mns1p is a relatively slowly acting enzyme (Jakob et al., 1998), and this trimming step is thought to act as a timer mechanism for glycoprotein folding, giving the protein a sufficient time window for obtaining its native conformation (Jakob et al., 1998; Helenius and Aebi, 2004). Once the protein has reached its native conformation, the Man_8_GlcNAc_2_ glycan acts as a determinant for cargo recruitment into COPII vesicles by the mannose lectins Emp47p and Emp46p (Sato and Nakano, 2002) (Figure 1B). Based on structural similarities to related human proteins and extensive testing of their glycan specificities, the α1-2 linked mannose of the A-branch was suggested to serve as binding motif for interaction with these mannose lectins (Satoh et al., 2006; Kamiya et al., 2008).

However, after trimming by Mns1p, a second mannose residue can be cleaved from the C branch by the α1-2 mannosidase activity of the Htm1p-Pdi1p complex (Pfeiffer et al., 2016). As a result of this reaction, Man_7_GlcNAc_2_ glycans containing an exposed terminal α1-6 linked mannose residue are formed (Clerc et al., 2009; Gauss et al., 2011; Pfeiffer et al., 2016). The Htm1p-Pdi1p complex preferentially demannosylates Man_8_GlcNAc_2_ glycans of partially unstructured polypeptides (Liu et al., 2016; Pfeiffer et al., 2016).

### Recognition of Endoplasmic Reticulum Associated Protein Degradation Substrates and Retrotranslocation

From three different ERAD systems, ERAD-L is responsible for eliminating ER luminal proteins which comprise also any secreted recombinant protein (Carvalho et al., 2006). The presence of a terminal α1-6 linked mannose residue of the Man_7_GlcNAc_2_ glycan is an important determinant for ERAD targeting of misfolded proteins (Quan et al., 2008; Kniss et al., 2018). However, also other glycans displaying a terminal α1-6 linked mannose, for example such forms as generated through *ALG3* deletion are recognized by Yos9p (Szathmary et al., 2005; Clerc et al., 2009) (Figure 1B).When this residue is displayed, it can be recognized by the lectin domain of Yos9p (Friedmann et al., 2002; Buschhorn et al., 2004). In addition, Yos9p has affinity for unstructured or hydrophobic regions of misfolded proteins (Smith et al., 2014). Thus, a bipartite signal requiring both aberrant protein structures and the α1-6 linked mannose signal is utilized for targeting of a glycoprotein to ERAD (Xie et al., 2009).

The HRD1 complex consists of the transmembrane proteins Hrd1p, Hrd3p, Usa1p, Ubx2p and Der1p and the cytosolic components Cdc48p, Npl4p and Ufd1p and mediates retrotranslocation into the cytosol and ubiquitinylation (Carvalho et al., 2006). Recognition by Yos9p leads to targeting of the misfolded protein towards the HRD1 complex (Martinez Benitez et al., 2011). It was suggested that Hrd3p recognizes sequences downstream of the glycosylation site that are not in their native state and Yos9p makes this more specific by only targeting misfolded glycoproteins to it (Gauss et al., 2006; Mehnert et al., 2015; Wu et al., 2020). The misfolded protein then associates with the close by Der1p, and it was proposed that this association leads to the unfolding and insertion of the substrate into substrate channel of the translocon complex that is formed by Hrd1p and Der1 (Carvalho et al., 2010; Mehnert et al., 2013; Vasic et al., 2020; Wu et al., 2020).

When the substrate emerges on the cytosolic side of the HRD1 complex, the ubiquitination step is performed by Hrd1p and this polyubiquitination prevents the slipping back of the protein into the ER lumen (Hiller et al., 1996; Friedlander et al., 2000; Bays et al., 2001; Metzger et al., 2013). When ubiquitinated, the substrate polypeptide is pulled away from the ER membrane by a complex of Cdc48p, Ufd1p and Npl4p (Wolf and Stolz, 2012) and it is directed to degradation by the 26S proteasome after removal of the N-glycans by Png1p (Suzuki et al., 2000; Hirsch et al., 2003).

### Glycoengineering Strategies in Yeasts

The humanization of the yeast N-glycans consists of two main objectives, first, to create suitable N-glycan structures that can act as substrates for the subsequent glycan maturation steps and, second, to introduce the required mammalian mannosidase and glycosyltransferase activities. Here, we focus on the steps and approaches leading to the formation of the simplest complex-type N-glycan GlcNAc_2_Man_3_GlcNAc_2_. Undesired and not well documented side effects of the glycoengineering are often growth defects that can stem from the approach chosen for generation of the glycan acceptor and from the introduction of the required glycan processing enzymes into the Golgi apparatus (Jacobs et al., 2009; Song et al., 2010; Parsaie Nasab et al., 2013).

In mammals, the first glycan processing steps taking place in the Golgi apparatus are the trimming of the remaining α1-2 linked mannoses from Man_8_GlcNAc_2_ by α-1,2-mannosidases IA, IB and IC (Bause et al., 1993; Tremblay et al., 1998; Tremblay and Herscovics, 2000). After mannosidase I trimming, the Man_5_GlcNAc_2_ glycan receives a β1-2 linked GlcNAc residue from GlcNAc transferase I (GnTI), forming a hybrid-type GlcNAcMan_5_GlcNAc_2_ glycan (Oppenheimer and Hill, 1981). The pathway towards complex-type glycans proceeds by trimming of the two remaining mannose residues from the B and C branches by α-mannosidases II and IIx (Tabas and Kornfeld, 1978; Misago et al., 1995; Shah et al., 2008). The α1-6 linked mannose of the GlcNAcMan_3_GlcNAc_2_ glycan receives a β1-2 linked GlcNAc residue from GlcNAc transferase II (GnTII), forming the complex-type GlcNAc_2_Man_3_GlcNAc_2_ glycan (Bendiak and Schachter, 1987). Depending on the sequence of these processing steps, Man_8_GlcNAc_2_, and Man_5_GlcNAc_2_ glycans can be selected as starting point for humanization of N-glycosylation pathway in the Golgi apparatus. Furthermore, from *in vitro* experiments it is known that GnTI can also act on Man_3_GlcNAc_2_ glycan substrates (Vella et al., 1984) and thus Man_3_GlcNAc_2_ glycans can serve as third road towards the formation of complex-type N-glycans.

**FIGURE 2:**
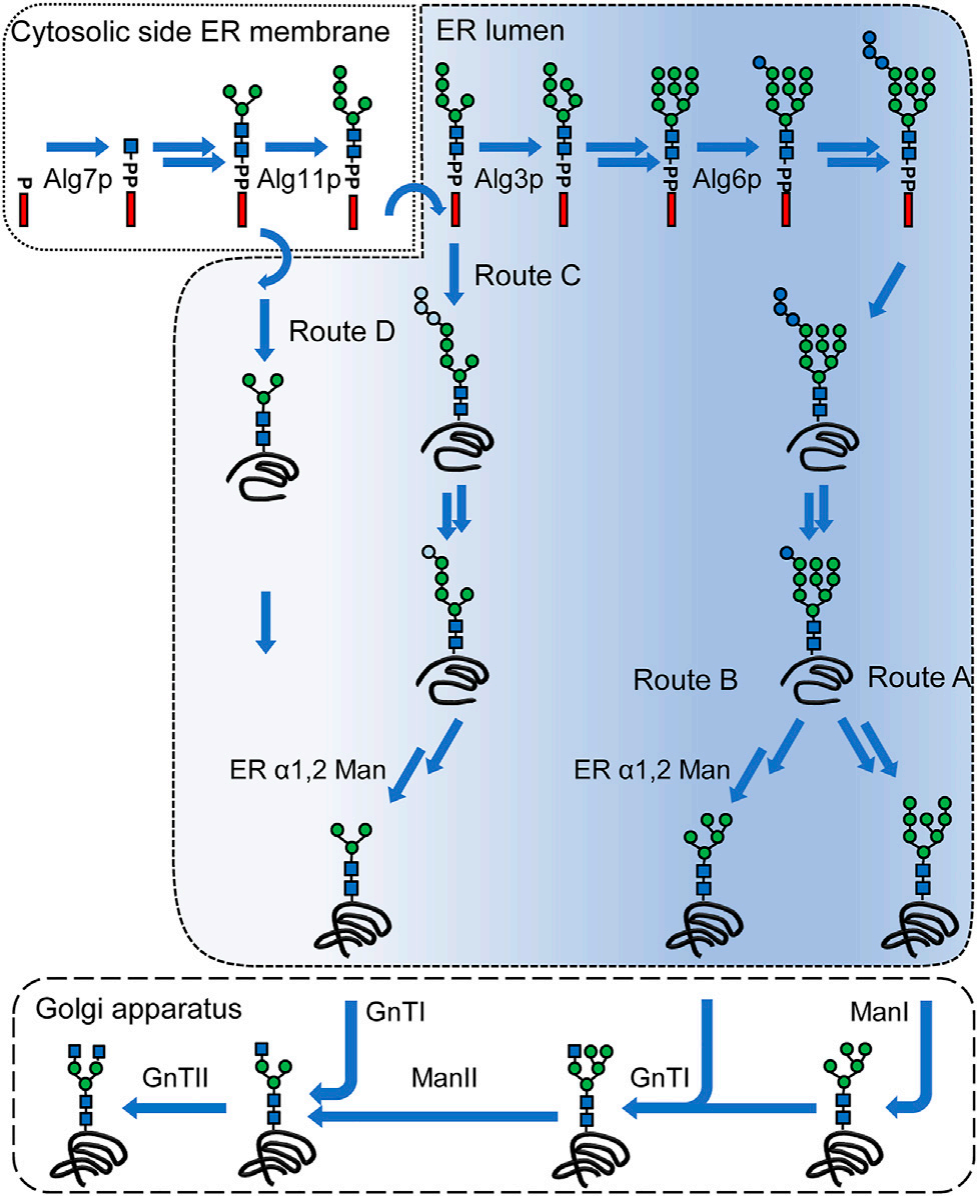
N-linked glycosylation pathways leading to complex-type N-glycans. A simplified lipid-linked oligosaccharide (LLO) biosynthesis pathway is depicted in the top of the figure. LLO synthesis is initiated on the cytoplasmic side of the ER membrane by Alg7p. A Man_5_GlcNAc_2_ structure is assembled on the cytoplasmic side by the consecutive action of the mannosyltransferases Alg13/14p, Alg1p, Alg2p and Alg11p. This structure is flipped into the ER lumen, where the modification of the LLO is continued by Alg3p. The B-branch is completed by the action of Alg9p after which the C-branch is started by Alg12p and completed by Alg9p. The triglucosyl cap is added by Alg6p, Alg8p and Alg10p generating a Glc_3_Man_9_GlcNAc_2_. This structure is transferred onto a protein by the oligosaccharyltransferase complex. Two glucose residues are removed and Glc_1_Man_9_GlcNAc_2_ mediates binding to calnexin. After release of the protein from calnexin and folding of the protein, the terminal mannose of the B-branch is removed (route A). N-glycans of glycoproteins undergo trimming and transfer reactions in the Golgi apparatus. First, α1,2 mannosidase activities (ManI) are trimming the α1,2 mannose residues, after which first GlcNAc residue is added by GnTI. The remaining mannose residues are trimmed off by ManII, which create the substrate for GnTII that adds a second GlcNAc residue. Route B) includes an α1,2 mannosidase activity in the ER that gives rise to the Man_5_GlcNAc_2_ glycan structure in the ER. This structure is modified in the Golgi apparatus by GnTI and route A and B converge. By deletion of *ALG3* gene, LLO biosynthesis is abrogated creating a Man_5_GlcNAc_2_ that can be glucosylated to varying extent and is transferred onto a protein (route C). After removal of glucose residues and possible interactions with calnexin, an α1,2 mannosidase activity converts this structure into a Man_3_GlcNAc_2_ structure. Alternatively, the Man_3_GlcNAc_2_ can be generated biosynthetically by deletion of *ALG11* and *ALG3* genes and this glycan is transferred onto a protein (route D). The Man_3_GlcNAc_2_ glycan structure directly serves as a substrate for GnTI and GnTII. The color gradient indicates the presence or absence of canonical glycan features involved in interaction with calnexin/calreticulin and Yos9p, respectively. Blue circles: glucose, green circles: mannose, blue squares: N-acetylglucosamine. Light blue circles indicate reduced glucosylation of the A-branch. Arrows without names: multiple steps; arrows with names: specific enzymes are marked.

Utilizing the Man_8_GlcNAc_2_ as the starting structure, the mammalian N-glycosylation pathway must be reproduced introducing mannosidase activities (ManI and ManII) and glycosyltransferases (GnTI and GnTII) into the Golgi apparatus (Figure 2, route A). By applying an extensive screening approach appropriate genetic constructs for optimal enzymatic activities were identified that led to a strain producing proteins with complex-type GlcNAc_2_Man_3_GlcNAc_2_ of very high homogeneity in *P*. *pastoris* (Hamilton et al., 2003).

Man_5_GlcNAc_2_ glycans formed in the ER can serve as the substrate for GnTI. This approach starts from native ER glycan forms but includes a heterologous α-1,2 mannosidase activity for the trimming of all α1-2 linked mannoses (Figure 2, route B). This strategy was first implemented in *S. cerevisiae*, where a HDEL-tagged α1-2 mannosidase from *Aspergillus saitoi* was expressed leading to formation of Man_5_GlcNAc_2_ structures and later adapted in *P*. *pastoris*, where the expression of an HDEL-tagged α-1,2 mannosidase from *Trichoderma reesei* eliminated over 85% of the α1-2 linked mannoses (Chiba et al., 1998; Callewaert et al., 2001). Using this strategy ManII, GnTI and GnTII activities are required in the Golgi apparatus and an increasing N-glycan heterogeneity was observed with each additional glycan processing step introduced (Jacobs et al., 2009).

In *S*. *cerevisiae* and *P*. *pastoris*, using the Man_8_GlcNAc_2_ and Man_5_GlcNAc_2_ structures as starting point required deletions of genes encoding interfering mannosyltransferases including Och1p, and in *S*. *cerevisiae* additionally the mannosyltransferase Mnn1p and phosphomannosyltransferase Mnn4p in order to increase homogeneity of the glycan structure (Chiba et al., 1998; Hamilton et al., 2003; Vervecken et al., 2004).

Alternatively, a Man_3_GlcNAc_2_ glycan can be generated in the ER. This can be achieved through deletion of *ALG3* gene and expression of a heterologous α1-2 mannosidase activity in the ER (Figure 2, route C). In this approach the α-1,6 linked mannose which serves as acceptor for GnTII is formed during LLO biosynthesis. In order to remove the A-branch, expression of HDEL-tagged versions of α-1,2 mannosidase from *A. saitoi* or of α-1,2 mannosidase from *T. reesei* were used and yielded glycoproteins predominantly containing the Man_3_GlcNAc_2_ glycan. This approach was implemented in *P. pastoris, H. polymorpha, Y. lipolytica, A. niger and A. nidulans,* respectively, however, was not effective in *K. marxiani* (Bobrowicz et al., 2004; Kainz et al., 2008; Oh et al., 2008; De Pourcq et al., 2012b; Lee et al., 2020; Anyaogu et al., 2021). Using this strategy, GnTI and GnTII activities are required in the Golgi apparatus.

Incomplete glucosylation of the A-branch, and thus hypoglycosylation, and inefficient glucosidase I and II trimming are associated with *ALG3* deletion in various yeasts (Verostek et al., 1993; Aebi et al., 1996a; Bobrowicz et al., 2004; De Pourcq et al., 2012a; Anyaogu et al., 2021). The low glycosylation site occupancy was tackled by the overexpression of glucosyltransferase *ALG6*, which was earlier shown to partially restore the glucosylation deficiency in *S. cerevisiae* (Burda et al., 1999; De Pourcq et al., 2012a), while the interfering glucose residues were eliminated by the overexpression of α-glucosidase II activities (De Pourcq et al., 2012a; Anyaogu et al., 2021). Alternatively, in *P. pastoris*, the low glycosylation site occupancy was compensated by overexpression of single-subunit OST from *Leishmania major* (Choi et al., 2012).

Direct biosynthetic generation of Man_3_GlcNAc_2_ glycan can be achieved by the deletion of *ALG3* and *ALG11* genes terminating the LLO biosynthesis at a very early stage. The Man_3_GlcNAc_2_ glycan contains a terminal α-1,3 linked and α-1,6 linked mannose residue, respectively, that serve as acceptor for GnTI and GnTII (Figure 2, route D). This strategy has been utilized in *H. polymorpha and S. cerevisiae* (Song et al., 2010; Parsaie Nasab et al., 2013; Piirainen et al., 2021).

Due to the strong modification of the LLO biosynthesis, measures to compensate for the resulting hypoglycosylation phenotypes are required. In both organisms this was achieved by improving the flipping of the LLO into the ER lumen and in *S. cerevisiae* by additionally overexpressing a single-subunit OST (Song et al., 2010; Parsaie Nasab et al., 2013). Furthermore, a number of mannosyltransferases compete with GnTI and GnTII for the Man_3_GlcNAc_2_ glycan in the Golgi apparatus leading to glycan structures with additional mannose residues that precluded the complete processing into complex type N-glycans and reduce glycan homogeneity (Song et al., 2010; Parsaie Nasab et al., 2013; Piirainen et al., 2016, 2021).

## Discussion

Most of the glycoengineering approaches developed for yeasts proceed via the formation of non-native, truncated glycan structures in the ER. To date it is unclear if and to which extent these deviating structures might impact the quality of the produced glycoproteins or affect their turnover by ERAD. Most of the studies on ER protein quality control systems were done with artificial folding-deficient glycoproteins. However, how about folding-proficient glycoproteins that would be ultimately produced in glycoengineered yeasts? Among these relevant proteins are antibodies, and the comparably smaller proteins erythropoietin, granulocyte-macrophage colony-stimulating factor, and interleukin 10 that were expressed in glycoengineered *P*. *pastoris* or *S*. *cerevisiae* (Hamilton et al., 2006; Li et al., 2006; Jacobs et al., 2009; Parsaie Nasab et al., 2013; Piirainen et al., 2021). Importantly, these glycoproteins do not depend on glycans for proper folding and can be manufactured either as aglycones, in glycosylation incompetent host cells such as *Escherichia coli* or *in vitro* systems (Higgins, 2010; Yin et al., 2012). Thus, it is plausible that the altered glycan structures do not directly lead to misfolding of these proteins. However, they might affect the folding kinetics and lead to undesired interaction with the ERAD system.

IgG molecules have been shown to interact with molecular chaperones, protein disulfide isomerases and peptidylprolyl isomerase activities. Moreover, UGGT, and small amounts of calnexin were detected in samples of immunoprecipitated IgG indicating the involvement of the calnexin cycle in the folding of IgG (Meunier et al., 2002). *In vitro* studies with yeast Cne1p and chemically unfolded egg yolk IgY carrying Glc_1_Man_7-9_GlcNAc_2_ structures indicated that Cne1p reduces aggregation (Xu et al., 2004a). Whether and to which extent the antibody folding process is dependent on Cne1p *in vivo* is unknown. Glycan structures formed in both glycoengineering approaches that are based on altering the LLO biosynthesis partially or completely bypass the calnexin-dependent protein folding (Figure 2, route C and D).

The exposed α1-6 linked mannose on the Man_7_GlcNAc_2_ glycan is the canonical glycan motif recognized by Yos9p (Szathmary et al., 2005; Clerc et al., 2009). The identical exposed α1-6 linked mannose is also generated by expression of α1,2 mannosidase activity in ER that trims glycans to a Man_5_GlcNAc_2_ structure but lacks the complete A-branch (Figure 2, route B). Whether this glycan is recognized by Yos9p is unknown. Alternatively, exposed α1-6 linked mannose residues, such as the one generated by *ALG3* deletion are recognized by Yos9p (Szathmary et al., 2005; Clerc et al., 2009). The identical α1-6 linked mannose is present in *ALG3* deletion expressing α1,2 mannosidase activity and *ALG11 ALG3* deletion strains, though the interaction of this glycan with Yos9p remains unknown (Figure 2, route C,D). Overall, the glycoengineering approaches have the potential to generate glycan structures that are recognized by Yos9p without the need for prior processing by Htm1p-Pdi1p complex.

What is the impact of ERAD system on the clearance of an IgG? In wild-type *S*. *cerevisiae*, deletion of ERAD components *HRD1*, *HRD3*, *YOS9*, *HMT1* and *UBC7* delayed IgG cellular clearance. Moreover, antibody clearance in an *ALG3* deletion strain with functional ERAD was comparable to the clearance in wild-type strain indicating that an exposed α1-6 linked mannose is sufficient for ER clearance (de Ruijter and Frey, 2015). Furthermore, deletion of *HRD1*, but not of *HTM1* or *YOS9* reduced clearance rates in an *ALG3 ALG11* deletion strain (Piirainen and Frey, 2020). This indicates that glycan-independent mechanisms additionally can contribute to IgG clearance in yeast.

In summary, the modifications introduced by glycoengineering into the N-glycan processing pathway have the potential to impair glycan-dependent protein folding, i.e., by by-passing calnexin and to generate glycan structure that are recognized by ERAD. However, our current understanding is incomplete and further studies will be required.
